# Behavioural adjustment of children with intellectual disability and their sibling is associated with their sibling relationship quality

**DOI:** 10.1111/jir.13006

**Published:** 2023-01-05

**Authors:** N. K. Hayden, R. P. Hastings, T. Bailey

**Affiliations:** ^1^ Centre for Educational Development, Appraisal and Research University of Warwick Coventry UK; ^2^ Centre for Developmental Psychiatry and Psychology, Department of Psychiatry Monash University Melbourne Australia

**Keywords:** autism, developmental disability, families, intellectual disability, relationships, siblings

## Abstract

**Background:**

Understanding sibling relationship quality is important, as it is associated with mental health outcomes in both childhood and adulthood. Arguably, these relationships are even more important for individuals with intellectual disability, as siblings can be important sources of care, support, advocacy and friendship for one another. The intellectual disability field, however, has a tendency to assume that the relationship lacks reciprocity, and that it is the sibling with intellectual disability who affects the sibling, and that this effect is somehow ‘negative’.

**Methods:**

We examined whether the behaviour problems and prosocial behaviour of 500 child sibling pairs, where one child has an intellectual disability, were associated with their sibling relationship quality. Measures included the Strengths and Difficulties Questionnaires and the Sibling Relationship Questionnaire. Family poverty, the gender of both children, birth order and whether the child with intellectual disability had autism or Down syndrome were also included in the analyses.

**Results:**

Confirmatory factor analysis indicated an adequate model fit for the latent variables measuring sibling relationships. The final structural model found that the prosocial behaviour and internalising problems of the children with intellectual disability, their typically developing siblings' prosocial behaviours and sibling birth order were associated with intimacy–companionship in the sibling relationship. The internalising, externalising and prosocial behaviours of the children with intellectual disability, their siblings' externalising behaviours and sibling birth order were associated with antagonism–quarrelling in the sibling relationship.

**Conclusions:**

We found that the behaviours of *both* the child with intellectual disability and their sibling were associated with *both* ‘positive’ and ‘negative’ dimensions of their sibling relationship. This indicates a bidirectional and reciprocal effect.

## Background

Intellectual disability is characterised by reduced intellectual function and reduced everyday adaptive skills with an onset before the individual's 18th birthday (AAIDD 2019). A meta‐analysis estimated that approximately 1% of the global population has an intellectual disability (10.37/1000 population, 95% CI 9.55, 11.18 per 1000 population; Maulik *et al*. 2011). Individuals with intellectual disability may also have other developmental disabilities, such as autism, Down syndrome, cerebral palsy, fragile X syndrome or (for children under 5 years of age) global developmental delay. Families of children with intellectual and developmental disabilities have been the focus of academic study for several decades. Empirical studies indicate that siblings may be at risk of worse psychological outcomes compared with other siblings (Hayden *et al*. 2019).

Siblings may become increasingly important in the lives of their siblings with intellectual disability. Improvements in the health and life expectancies of disabled people, along with decreases in state welfare, have highlighted uncertainties about future care and support for people with intellectual and developmental disabilities (Power and Bartlett 2018). Although the future role of siblings is uncertain, researchers have speculated that siblings may be called upon to take on support and care roles (Leane 2019). There is an association between siblings taking on carer roles for their brothers and sisters with intellectual and developmental disabilities, and their sibling relationship quality. Closer sibling relationships may increase the likelihood that the sibling without an intellectual or developmental disability will be willing to take on this caring role in the future (Burke *et al*. 2012). Therefore, understanding and fostering sibling relationships in this group is vital from a policy and practice perspective, as sibling relationship quality may predict future sibling caregiving.

Understanding sibling relationships is important for both siblings. Sibling relationships potentially last from early childhood into old age, with siblings influencing one another's lives across the lifespan. Children learn and develop social behaviours within their sibling relationships that impact their wider relationships beyond the family and beyond childhood (Mandleco and Webb 2015). In the general population, research indicates that poorer sibling relationship quality is associated with poorer mental health outcomes, such as depression in adulthood and childhood, adjustment problems, internalising and externalising behaviours and substance abuse (Waldinger *et al*. 2007; Feinberg *et al*. 2012). Sibling relationships may be particularly salient in families of individuals with intellectual or developmental disabilities. Richardson and Jordan (2017) emphasised the way in which sibling relationships are ‘imperative to the lives of people with disabilities’ (Richardson and Jordan 2017, p. 1536). Many disabled people continue to face exclusion and discrimination in the community and in wider society. Sibling relationships, therefore, may provide an important source of support and friendship for disabled people.

The current research is informed by family systems perspectives. Family systems perspectives explore the way in which family members may influence and relate to one another (Cox and Paley 1997; White and Klein 2002; Hayden and Hastings 2022). The intellectual and developmental disability family research field tends to assume that it is the disabled child who will have a ‘negative’ effect on the rest of the family (Hastings 2016). There are data supporting a (relatively small) potential negative impact of living with a disabled brother or sister on siblings' well‐being (Hayden *et al*. 2019; Marquis *et al*. 2019). Recent population‐based studies have examined the psychological outcomes of both siblings of children with intellectual disability (Hayden *et al*. 2019) and developmental disabilities (Marquis *et al*. 2019) in comparison with siblings of children without disabilities. However, such research focused on individual family members does not fully reflect systems thinking (Rosenblatt 1994), and a more complete understanding is needed through the exploration of relationship outcomes, such as by exploring the sibling dyad. Systems thinking encourages us to conceptualise the sibling–dyad level relationship as reciprocal and interconnected in nature (Cox and Paley 1997; Cox 2010; Hayden and Hastings 2022) and to move beyond focusing entirely on the effect of the disabled child on their siblings.

Existing quantitative research on sibling relationships often includes measures of relationship dimensions of warmth, closeness, conflict, status/power and rivalry (Furman and Buhrmester 1985). These dimensions were developed by Furman and Buhrmester (1985) to avoid the ‘negative’/‘positive’ divide of relationship dimension scales prior to this measure. For example, a sibling relationship, using their measure, can be understood as both highly warm and highly conflictual, whereas prior relationship scales would measure warmth and conflict together to define a relationship as warm *or* conflictual. Therefore, this conceptualisation allows various dimensions of the relationship to be understood, and it allows for ambivalence, as well as detachment and disinterest by either sibling in the sibling relationship.

In the non‐disability literature, a meta‐analysis found that where children's and adolescents' sibling relationship quality had more warmth and less conflict, this was associated with lower internalising and externalising problem scores for participants (Buist *et al*. 2013). Therefore, sibling relationships are an important predictor for understanding children's and adolescents' behavioural and emotional outcomes. Studies exploring sibling warmth and closeness when one has a disability have found particularly positive sibling relationships, for example, when one sibling has Down syndrome (Hodapp and Urbano 2007), and this may be related to relatively elevated levels of prosocial behaviours in individuals with Down syndrome. Although many researchers have hypothesised that sibling relationships where one child has an intellectual or developmental disability might be more ‘negative’, the empirical evidence base does not substantiate this. Existing research exploring more ‘negative’ aspects of the sibling relationship have found similar levels of conflict between typically developing sibling pairs compared with sibling pairs where one has intellectual disability (Doody *et al*. 2010). Other studies have found that sibling pairs where one has an intellectual disability may have less conflict in their sibling relationships than other siblings (Kaminsky and Dewey 2001; Floyd *et al*. 2009). Existing autism research has identified that the behavioural problems of the child with autism has been associated with less warmth, closeness and more conflict in the sibling relationship (Hastings and Petalas 2014) or poorer sibling relationship quality (Jones *et al*. 2019). Therefore, there may be some variation in sibling relationship quality depending on whether the sibling with intellectual disability also has co‐occurring developmental disabilities, such as autism.

There have been fewer studies examining sibling relationship quality and outcomes for *both* siblings when one has an intellectual or developmental disability. We have identified only three such studies. Begum and Blacher (2011) found that conflict in the sibling relationship was associated with internalising behaviour problems in the non‐disabled sibling and externalising behaviour problems in the sibling with intellectual disability. Orsmond *et al*. (2009) found that sibling relationships were more positive when autistic siblings had fewer behaviour problems. The behaviours of siblings of children with Williams syndrome were also found to be associated with their sibling relationship quality (Cebula *et al*. 2019). Family systems perspectives would suggest that both siblings' behavioural and emotional problems would have an association with their sibling relationship quality and that these effects would be reciprocal. This lack of intellectual or developmental disability literature examining the effects of the behaviours of both siblings on their sibling relationship reveals significant assumptions that need to be challenged. These are (1) the tendency to assume that sibling relationships where one sibling has intellectual disability lack reciprocity, (2) that it is the sibling with intellectual disability who effects the sibling without an intellectual or developmental disability and (3) that this effect is somehow a ‘negative’ effect (cf. Hastings 2016). These assumptions are illustrated by the dominance of psychological outcomes studies in the intellectual or developmental disability sibling literature.

The primary aim of the current study was to explore whether, in sibling dyads where one has intellectual disability, the behavioural adjustment of both siblings is associated with their sibling relationship quality. We expected that both siblings' behavioural and emotional problems and/or prosocial behaviour would be associated with sibling relationship quality.

## Methods

### Participants

Participants were 500 primary parental caregivers reporting on nearest‐in‐age sibling dyads where one has intellectual disability, and both were aged between 4 years and 15 years and 11 months. Most of the primary caregivers were maternal figures (94.0% were female; and 89.0% were biological mothers). Table 1 summarises descriptive statistics for the sample included in the current study.

**Table 1:** Sample descriptive statistics

Demographic and family descriptive statistics	
Mean age of sibling in years (*SD*)	9.23 (3.12)
Mean age of child with intellectual disability in years (*SD*)	8.66 (2.68)
Sibling is older	56.7%
Sibling is male	47.0%
Sibling has a long‐standing illness, disability or infirmity	13.6%
Child with intellectual disability is male	67.1%
Child with intellectual disability has severe/profound intellectual disability	49.0%
Child with intellectual disability has autism	50.6%
Child with intellectual disability has Down syndrome	17.6%
Primary caregiver is female	94.0%
Primary caregiver is the biological mother	89.0%
Family has no indicators of poverty on the poverty composite. Poverty indicators:	26.2%
Family would struggle to raise £2000 in an emergency	48.4%
Family indicated that they were struggling financially	11.5%
Lowest quintile for neighbourhood deprivation	14.7%
Household is below the median for weekly household income	62.5%
Primary caregiver is White	93.1%
Primary caregiver is Asian	3.4%
Primary caregiver is Black	2.0%

### Measures

#### Behavioural and emotional adjustment

Participants completed the Strengths and Difficulties Questionnaire (SDQ; parent version; Goodman 1997) to measure the emotional and behavioural adjustment of both the children with intellectual disability and their nearest‐in‐age sibling. The SDQ is a 25‐item screening questionnaire with items rated on a 3‐point scale: *not true*, *somewhat true* or *certainly true*. Three SDQ scores were used: internalising problems (emotional and peer problems), externalising problems (conduct and hyperactivity problems) and prosocial behaviours. Goodman *et al*. (2010) argued internalising/externalising domains should be used in community samples and where users are not screening for disorder because these scores have more satisfactory convergent and discriminant validity than the individual SDQ subscales in community contexts. The SDQ has also been shown to have good validity for identifying behavioural and emotional problems in children with intellectual disability (Murray *et al*. 2020). In the current study, for the SDQ responses about the children with intellectual disability, the internal consistencies (Cronbach's α) for internalising problems was .77, externalising problems was .73, and prosocial behaviour was .85. For the SDQ responses about the siblings, the internal consistencies (Cronbach's α) for internalising problems was .83, externalising problems was .86, and prosocial behaviours was .85.

#### Sibling relationship quality

**Figure 1:**
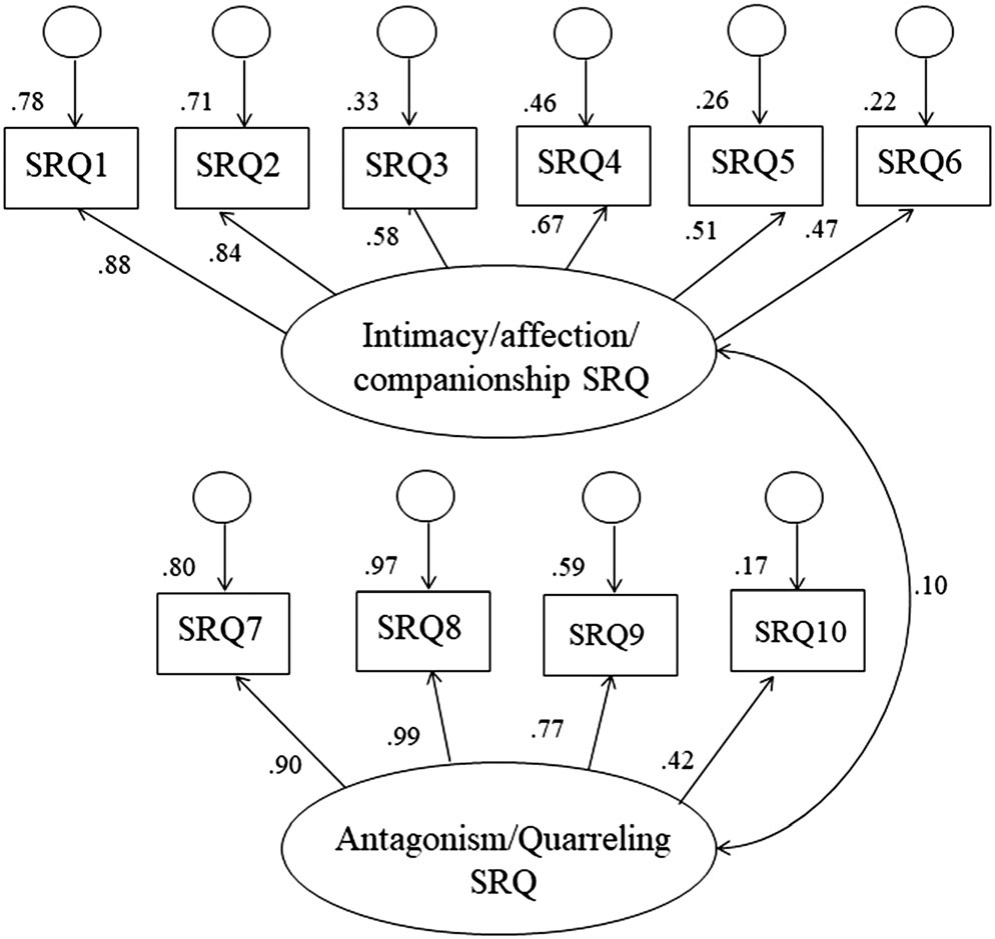
Initial measurement model, latent constructs measuring sibling relationship quality. *Note*: Key for each SRQ item with parentheses indicating the corresponding item number on the full, original SRQ measure: Intimacy: SRQ items 1 (44) and 2 (28) = talking and sharing private feelings; Companionship: SRQ items 3 (9) and 4 (25)—shared activities; Affection: SRQ items 5 (8) and 6 (24) = feelings of love and care; Quarrelling: SRQ items 7 (16) and 8 (32) = quarrelling and arguments; Antagonism: SRQ items 9 (10) and 10 (26) = insulting, name calling and mean behaviour

**Figure 2:**
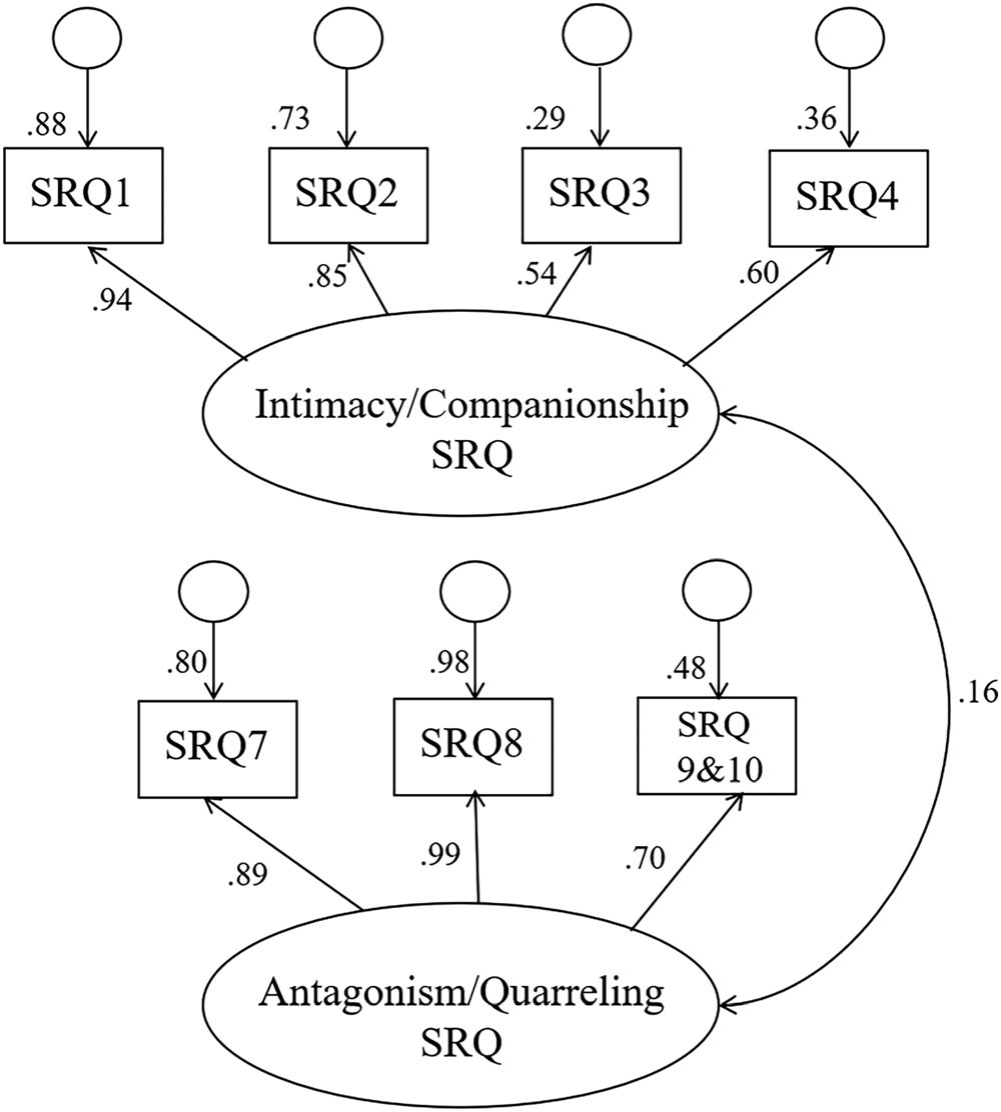
Final measurement model, latent constructs measuring sibling relationship quality

Items from the Sibling Relationship Questionnaire (SRQ; short form; Furman and Buhrmester 1985) were used to assess relationship quality in the sibling dyads. Although the short form SRQ has 39 items, 10 items were included in the survey to reduce participant burden. Two latent constructs were derived from these 10 items (see Figs 1,2). Primary caregivers respond to all statements about the sibling relationship on a 5‐point scale: *hardly at all*, *not too much*, *somewhat*, *very much* or *extremely much*. Internal consistency (Cronbach's α) in this sample was .84 for intimacy, companionship and affection and .84 for quarrelling and antagonism in the sibling relationship.

### Procedure and study design

Data for this study were from the first wave of the 1000 Families Study, a UK‐based, large‐scale, ongoing longitudinal data set of families with at least one child with intellectual disability living full‐time in the household (Hastings *et al*. 2020). The overall study sample size at wave 1 included 1184 primary caregivers. The inclusion criteria for the overall study required that families had: (1) at least one child aged between 4 years and 15 years and 11 months with an intellectual disability as self‐reported by their primary caregiver; (2) had at least one primary caregiver that consented and responded to the survey; and (3) were living within the UK. For this specific analysis, we then removed families where the child with intellectual disability did not have a sibling within the age range (4 years and 15 years 11 months old) or those with missing outcomes (*n* = 572 families removed) and those where the sibling themselves also had an intellectual or developmental disability (*n* = 112 families removed). Our study sample therefore consisted of 500 sibling pairs where one had intellectual disability and one did not have an intellectual or developmental disability.

Families were recruited using a multi‐point method: through websites and social media via UK research and charity organisations' newsletters and through special schools. Most participants completed the survey online, although to remove barriers to participation, postal surveys could be requested. Participants did not receive payments for taking part in wave 1 of this study.

The study received full research ethics approval. Participants provided fully informed consent. Best‐practice confidentiality and data protection standards were adhered to throughout.

### Analysis procedure

Structural equation modelling (SEM) was employed. SEM benefits from being theoretically driven (Kline 2016). Analyses were conducted using IBM SPSS AMOS 26 using maximum likelihood estimation, and a two‐stage process was undertaken. First, confirmatory factor analysis (CFA) was used to ascertain whether the SRQ items sufficiently measured latent constructs related to sibling relationship quality. Further adjustments to the measurement model were required to improve model fit and the loading of items in relation to the latent constructs. Fit was measured using statistics available from models where means and intercepts were estimated to account for missing data and included the Tucker–Lewis index (TLI; ≥.95), the comparative fit index (CFI; ≥.95) and the root mean square error of approximation (RMSEA; ≤.06;). The cut‐off levels indicated were recommended by Hu and Bentler (1999) and were subsequently endorsed by Cabrera‐Nguyen (2010).

Structural models were then fitted to examine the main research question exploring associations between the behaviours of the siblings and the children with intellectual disability with their sibling relationship quality. Variables were included in the models iteratively using a forward entry approach. First, we incorporated the SDQ internalising, externalising and prosocial behaviour scores—for the children with intellectual disability and their siblings—with the latent constructs encapsulating sibling relationship quality. The second structural model included control variables in one model to identify which variables were significantly associated with sibling relationship quality. Control variables were identified from existing research: sibling birth order (Braconnier *et al*. 2018), both children's gender (Orsmond and Seltzer 2000; Cuskelly and Gunn 2003; Floyd *et al*. 2016), family poverty (income poverty; subjective poverty; ability to raise emergency funds and; neighbourhood deprivation—see Table 1) (Emerson 2003; Emerson 2004; Hayden *et al*. 2019) and whether the children with intellectual disability also had Down syndrome, or autism (as existing studies suggest that sibling relationship quality may be affected by whether the disabled sibling has autism vs. Down syndrome; Hodapp and Urbano 2007; Orsmond and Seltzer 2007). The final structural model incorporated each of the control variables and behaviour variables that met the threshold of *P* < .10 for their association with the sibling relationship latent constructs.

## Results

### Measurement models

Figure 1 illustrates the initial measurement model for the 10 items from the SRQ as two latent constructs (intimacy, affection and companionship and antagonism–quarrelling). The model fit was not sufficient (χ^2^ (34) = 834.46; *P* < .001; CFI = .74; TLI = .58; RMSEA = .22).

Alternative models and associations were explored to improve the model fit including a unidimensional model, removing items with low factor loadings and aggregating items (i.e. parcelling; Matsunaga 2008). Parcelling has the benefit of improving model fit, stabilising parameter estimates as well as offering potential psychometric benefits (Matsunaga 2008).

The final measurement model is shown in Figure 2. For the latent construct measuring intimacy, affection and companionship, the items measuring affection—items five and six—were removed from the measurement model as they had the lowest factor loadings (.51 and .47 in Figure 1, respectively). For the latent construct measuring antagonism–quarrelling in the sibling relationship, parcelling was used to improve the model fit of the construct. Both the ninth and tenth items were capturing ‘antagonism’ in the sibling relationship, and these two items were parcelled to better distil the latent construct (Matsunaga 2008). These changes improved the model fit (χ^2^ (13) = 89.85; *P* < .001; CFI = .96; TLI = .92; RMSEA = .11).

### Structural models

The structural models exploring predictors of sibling relationship quality are summarised in Table 2. The first stage involved identifying the SDQ scores of the child with intellectual disability and their sibling that were (*P* < .10) associated with sibling relationship quality (see Table 2; models 1.0 and 1.1.). This stage confirmed that the child with intellectual disability's prosocial behaviour and internalising problems and the sibling's prosocial behaviour were associated with intimacy–companionship. Model 1.1 (χ^2^ (32) = 83.72; *P* < .001; CFI = .98; TLI = .96; RMSEA = .06) also confirmed that the child with intellectual disability's prosocial behaviour and externalising and internalising problems and the sibling's externalising problems were associated with antagonism–quarrelling. Other pathways were removed from the final structural models at this stage. All the retained pathways were statistically significant (*P* < .001).

**Table 2:** Structural models predicting sibling relationship quality

Structural models	χ^2^ (*df*)	Model *P*	CFI	TLI	RMSEA	All paths *P* < .10
**1.0** All behaviours of child with intellectual disability and sibling.	86.12 (33)	<.001	.98	.95	.06	No
**1.1** Behaviours of child with intellectual disability (int., ext., pro.); sibling (ext., pro.)	83.72 (32)	<.001	.98	.96	.06	Yes
**2.0** All control variables	163.57 (49)	<.001	.95	.89	.07	No
**2.1** Control variables: sibling birth order, sibling gender, autism, poverty	154.57 (37)	<.001	.94	.90	.08	Yes
**3.0** Significantly associated behaviours of child with intellectual disability, sibling and control variables (see variables included in models 1.1 and 2.1)	221.49 (65)	<.001	.95	.89	.07	No
**3.1** Final model (Figure 2): sibling: int., pro., child with intellectual disability: ext., int., pro., and sibling birth order	199.00 (47)	<.001	.95	.89	.08	Yes *p* < .05
*Notes*: All behaviours included: Int., SDQ internalising behaviours. Ext., SDQ externalising behaviours. Pro., SDQ prosocial behaviours. All control variables included: single‐ or two‐parent household, poverty composite, sibling birth order, child with intellectual disability gender, sibling gender, whether the child with intellectual disability had autism, whether the child with intellectual disability had Down syndrome.						

The second stage involved identifying the control variables that would be retained in the final structural models by removing those with associations with a *P* value > .10. Table 2 provides a summary of this stage (models 2.0 and 2.1). Model 2.1 (χ^2^ (37) = 154.57; *P* < .001; CFI = .94; TLI = .90; RMSEA = .08) was used to identify included control variables.

The final structural models are summarised in Table 2 (models 3.0 and 3.1). Further control variables were removed after model 3.0 (χ^2^ (65) = 221.49; *P* < .001; CFI = .95; TLI = .89; RMSEA = .07) where the *P* value for some of the associations was >.05.

**Figure 3:**
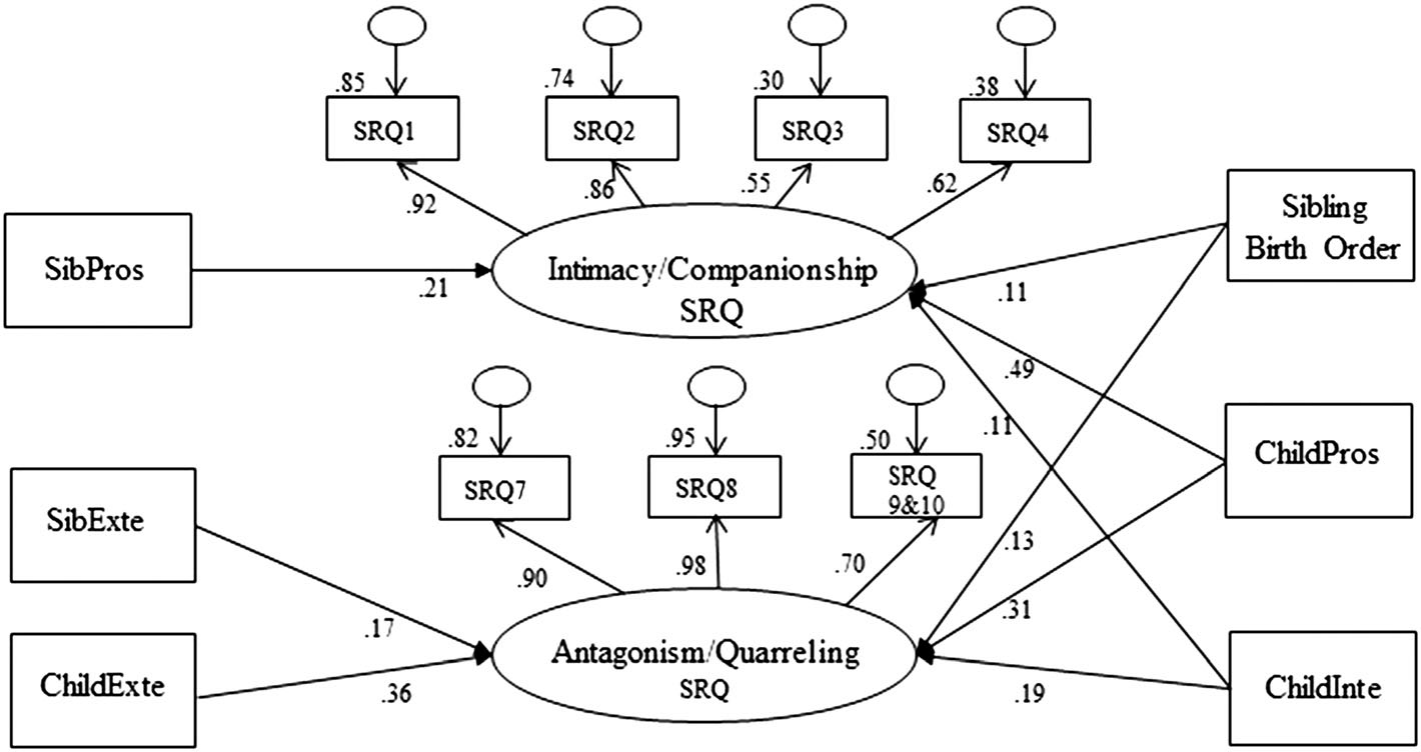
Final structural model: associations between the behaviours of sibling pairs and their sibling relationship quality where one has intellectual disability. Child Exte, child with intellectual disability externalising behaviours SDQ score; ChildInte, child with intellectual disability internalising behaviours SDQ score; ChildPros, child with intellectual disability prosocial behaviours SDQ score; SibExte, sibling externalising behaviours SDQ score; SibPros, sibling prosocial behaviours SDQ score.

The model fit for the final structural model (model 3.1 in Table 2) was adequate (χ^2^ (47) = 199.00; *P* < .001; CFI = .95; TLI = .89; RMSEA = .08). Figure 3 provides a visual summary of the final structural model, as well as standardised estimates of the associations between the variables and latent constructs measuring sibling relationship quality. Table 3 provides these standardised estimates and the *P* values for these associations.

**Table 3:** Final structural model associations between predictors and sibling relationship quality latent constructs

Predictors of sibling relationship quality	Association with intimacy–companionship in the sibling relationship standardised regression weights (*P value)*	Association with antagonism—quarrelling in the sibling relationship standardised regression weights (*P value)*
Sib externalising behaviours	‐	.17 (<.001)
Sib prosocial behaviours	.21 (<.001)	‐
Child with intellectual disability internalising behaviours	.11 (<.011)	.19 (<.001)
Child with intellectual disability externalising behaviours	‐	.36 (<.001)
Child with intellectual disability prosocial behaviours	.49 (<.001)	.31 (<.001)
Sib birth order	.11 (.013)	.13 (.001)

The child with intellectual disability's prosocial behaviour had the strongest positive association with intimacy–companionship in the sibling relationship (β = .49, *P* < .001). The sibling's prosocial behaviour (β = .21, *P* < .001) and the child with intellectual disability's internalising problems (β = .11, *P* = .011) were also positively associated with intimacy–companionship. The child with intellectual disability's externalising problems had the strongest association with antagonism–quarrelling in the sibling relationship (β = .36, *P* < .001), and their prosocial behaviour (β = .31, *P* < .001) and internalising problems (β = .19, *P* < .001) were also significant paths in the model, along with the siblings' externalising problems (β = .17, *P* < .001). For sibling birth order, where the sibling was younger than their sibling with intellectual disability, this was associated with both more intimacy–companionship (β = .11, *P* = .013) and more antagonism–quarrelling (β = .13, *P* = .001).

## Discussion

We explored whether the behavioural and emotional adjustment of children with intellectual disability and their closest‐in‐age sibling was associated with their sibling relationship quality. The final structural model (Fig. 3) showed that the behaviour of *both* the children in the sibling dyad was associated with sibling relationship quality. This finding, in the context of broader general sibling literature, is expected. However, this is an important finding in the intellectual disability‐specific sibling literature, where researchers may have tended to assume a lack of reciprocity in the sibling relationship and a negative one‐way direction of effect from the child with intellectual disability to their sibling.

Our finding that there was an association between the prosocial behaviour of the sibling with intellectual disability with both the antagonism–quarrelling *and* intimacy–companionship domains may appear counterintuitive. However, children with intellectual disability with higher levels of prosocial behaviours may have milder intellectual disability and therefore may have more social skills. Children with intellectual disability with higher social skills may be able to engage with their siblings in a more equal way—and *vice versa—*with the sibling treating their siblings with intellectual disability more equally/typically, including in terms of antagonism–quarrelling. In this way, this conceptualisation of sibling relationships is not a binary of ‘positive’ and ‘negative’, nor is it a spectrum with antagonism–quarrelling on one side and intimacy–companionship on the other. Siblings will experience both, sometimes simultaneously, in their relationships with one another.

The only covariate that remained in the final structural model was sibling birth order. Where the sibling without intellectual or developmental disability was younger than their sibling with intellectual disability, this was associated with both more intimacy–companionship and more antagonism–quarrelling in the sibling relationship. This may be related to the way in which younger siblings may be more likely to spend time with and treat their sibling with intellectual disability more equally, both arguing and playing together more freely. Younger siblings are likely to be at a closer developmental stage to their sibling with intellectual disability compared with older siblings and are therefore more likely to enjoy doing similar things together. Younger siblings are also less likely to have self‐control over their own behaviours compared with older siblings and so are more likely to engage in quarrelling and antagonism with their siblings with intellectual disabilities. Siblings spending more time together is likely to be associated with both more intimacy–companionship and antagonism–quarrelling—as there are more opportunities for engagement in all its forms. Older siblings may take on more caring or responsibility‐based roles, whereby they disengage or avoid antagonism–quarrelling with their siblings with intellectual disabilities. Older siblings may be more mature and therefore have more self‐control, enabling siblings to reduce antagonism–quarrelling in their relationship with their siblings with intellectual disabilities. However, this avoidance may come at a cost, reducing the intimacy‐companionship in their relationship with their younger sibling with intellectual disability. Overall, the sibling relationship dimensions of intimacy–companionship and antagonism–quarrelling are not binary constructs. All relationships, but especially sibling relationships, are not intimate and companionable *or* quarrelsome and antagonistic; they can be both and neither to varying degrees at different times.

In terms of theoretical implications, these findings support a family systems perspective whereby both siblings' behaviours (both the more ‘negative’ and the more ‘positive’ aspects) have an association with both ‘positive’ and ‘negative’ aspects of the sibling relationship. Although family systems perspectives can be used to model reciprocal associations about sibling relationships when one has intellectual disability such as in this study, it is interesting that family systems perspectives are rarely applied in this way, but rather in an arguably over‐simplified way that assumes a negative narrative. It may be valuable to consider the non‐disability sibling literature, which favours social learning theory (Feinberg *et al*. 2012) over family systems perspectives to theorise and model the way siblings' relationships with one another are associated with both siblings' behaviours. Social learning theory assumes that both siblings learn and affect one another in an equal way, emphasising reciprocity. It may be worth considering why this theory is applied commonly in the general sibling literature, but not generally in the intellectual or developmental disability and disability sibling literature. One reason may be that sibling intellectual or developmental disability researchers have assumed a negative, one‐sided and non‐reciprocal narrative about siblings of children with intellectual or developmental disability. Reciprocity is the essential premise of social learning theory, whereas for family systems perspectives, it is one component of an overall concept (Hayden and Hastings, 2022). Future research should seek to ensure that family systems perspectives are not used simply to justify a focus on siblings' outcomes but, rather, are engaged meaningfully to inform research design, analyses and interpretations of findings.

### Limitations

A limitation of the current study is that the models were from cross‐sectional data. Longitudinal data would be required to provide further support to the final model and importantly, to ascertain the direction of the pathways beyond the essentially theoretical model provided by these cross‐sectional data. The final structural model presented is also only indicative at this stage due to its ‘adequate’ model fit. Therefore, other variables or model configurations may explain and fit the data better.

The data included in this study were from primary caregiver report only. We know that parents and children report on children's sibling relationships differently (Rankin *et al*. 2017; Cebula *et al*. 2019). For example, a child sharing a toy with their sibling may score highly in prosocial behaviours as perceived by a parent, but if the reason for their sharing is to avoid arguments, this may be indicative of avoidant behaviours (perhaps associated with internalising problems) that are not being identified as such by their primary caregiver. Similarly, quarrelling or antagonism may be helpful ways for children to communicate their needs and frustrations or to learn from siblings about how to manage human relationships more generally. Another limitation is that the sample is not representative, for example, most of the primary caregivers in this sample were White British.

The measurement model for sibling relationship quality measured using the SRQ required items to be parcelled and dropped to improve item loadings onto the latent constructs and to improve the model fit of the latent constructs. Even after improvements, the model fit indicated the model was adequate. Therefore, alternative measures of sibling relationship quality should be explored in future. Measuring something as ambivalent and dynamic as sibling relationship quality, particularly from the perspective of someone outside of that relationship (i.e. caregiver reported) is challenging enough before we begin to also consider the associated measurement challenges when one of those children in the sibling dyad has an intellectual disability. For example, items surrounding quarrelling may have been difficult for some primary caregivers to respond to if their child with intellectual disability was minimally verbal. The model fit also has conceptual problems. We were essentially left with a construct that measured ‘positive’ and ‘negative’ dimensions of sibling relationship quality, which lacks nuance.

### Future research directions

The limitations outlined provide directions for further research. For example, longitudinal data would be required to further support the model presented in this study to help us further understand the direction of the pathways and to understand how sibling relationships change over time. Future research should consider if there are more effective ways of measuring sibling relationships where one sibling has intellectual disability. This could involve providing further guidance to primary caregivers about how to interpret the items on the SRQ where one child has an intellectual disability, or this may require the development of a new questionnaire specifically for child sibling relationships where one sibling has an intellectual disability. The only covariate that remained in our final model was whether non‐disabled siblings were older or younger than their siblings with intellectual disabilities. Therefore, future studies may also consider expanding age‐related analyses, such as how the magnitude of the age gap may affect the sibling relationship.

In future, researchers should also find ways to incorporate self‐report responses, not just from the non‐disabled sibling but also from their sibling with intellectual disability. Qualitative research would be an important way of developing methods to incorporate both siblings' perspectives on the sibling relationship, with the flexibility to be appropriate for the differences and preferences of both siblings. Finally, future studies may explore models that incorporate functioning across multiple family members, as well as models conceptualised at the sibling‐dyad level. For example, models could include family and parental conflict, differential parenting and parental modelling and skills. Applying theoretical frameworks such as family systems perspectives and social learning theory may inform complex, reciprocal, dyad‐level studies.

### Practical and clinical implications

Any intervention developed to improve relationship quality at the level of the sibling dyad ought to be cognizant of, and involve, the full family system. This approach is supported by data exploring associations between sibling behaviours and relationships with parenting practices, and sibling relationships with marital relationships (cf. Feinberg *et al*. 2012; McHale *et al*. 2012). The final structural model in this study provides some information that may be used to develop interventions to support sibling relationship quality when one sibling has an intellectual disability. For example, prosocial behaviour from both siblings was associated with sibling relationship quality, suggesting an intervention that focused on increasing prosocial behaviour in both the child with intellectual disability and sibling may be fruitful. The importance of birth order in the final structural model also supports a differential approach depending on whether the sibling is older or younger than their sibling with intellectual disability. For example, where the sibling is older than their sibling with intellectual disability, interventions may explore facilitating more contact between the siblings. Whereas when the sibling is younger than their sibling with intellectual disability, support may take the form of facilitating conflict management in the sibling dyad.

## Source of funding

This work was funded by an Economic and Social Research Council doctoral scholarship (ES/J500203/1). This research has also been part‐funded by the UK charity Sibs. The 1000 Families Study was funded by Cerebra.

## Conflict of interest

We have no conflicts of interest to declare.

## Ethics statement

Full ethical approval was granted for this study from the UK National Health Service (NHS) West Midlands—South Birmingham Research Ethics Committee: REC reference number: 15/WM/0267 (11 September 2015). An amendment was granted to extend the age range (7 March 2017). An amendment was granted for changes made to wave 2 (8 February 2018), and the ethics committee was informed of further minor changes made to the documents for wave 2 (27 February 2018). Sponsor approval was also obtained from the University of Warwick. Participants provided informed consent to take part in the 1000 Families Study and for findings from the 1000 Families Study to be published.

## Data Availability

No data are available. Data from this study are not available for sharing due to ethical approval requirements. Researchers interested in collaboration should contact the authors with their expression of interest.
